# Anti-Epileptic Drug Toxicity in Children

**DOI:** 10.3390/children5050057

**Published:** 2018-05-01

**Authors:** Imti Choonara

**Affiliations:** Academic Division of Child Health, The Medical School, University of Nottingham, Derbyshire Children’s Hospital, Derby DE22 3DT, UK; imti.choonara@nottingham.ac.uk

**Keywords:** drug toxicity, antiepileptic drug, behavioural problems, valproate, lamotrigine, levetiracetam

## Abstract

Anti-epileptic drugs (AEDs) have had a major impact on children, improving their quality of life and significantly reducing both morbidity and mortality. They are, however, associated with significant toxicity. Behavioural problems and somnolence are the most frequent adverse drug reactions for many AEDs. Unfortunately, the comparative risk of drug toxicity for different AEDs has been inadequately studied. Drug toxicity is poorly reported in randomised controlled trials. Prospective cohort studies are the best way to study drug toxicity. There have been a few prospective cohort studies of children with epilepsy, but the numbers of children have been small. Systemic reviews of the toxicity of individual AEDs have been helpful in identifying the risk of drug toxicity. Parents of children with epilepsy and the children and young people who are due to receive AED treatment have the right to know the likelihood of them experiencing drug toxicity. Unfortunately, the evidence base on which health professionals can provide such information is limited.

Epilepsy is the most common neurological disorder in children, affecting approximately 1 in 200 children. Anti-epileptic drugs (AEDs) have had a major impact on children, improving their quality of life and significantly reducing both morbidity and mortality. Carbamazepine and sodium valproate are the most widely used old-generation AEDs [1]. Phenobarbital is used more frequently in Asia [1]. New-generation AEDs, in particular, lamotrigine, levetiracetam, and topiramate, are being used more frequently [1]. A recent large study in the USA suggested that levetiracetam was the most widely-used AED in children with epilepsy, with more than one in four children receiving levetiracetam [2]. Monotherapy is the recommended treatment as it minimises adverse drug reactions (ADRs) and a systematic review showed that the majority of children with epilepsy received monotherapy [1].

Unfortunately, AEDs are associated with significant toxicity. In the majority of cases ADRs are mild. It is worth noting, however, that AEDs were the most common cause of drug induced fatalities in children in the UK [3]. Over a period of 36 years, there were 65 fatalities reported in association with AED use [3]. Prospective studies have suggested that between one third and two thirds of children receiving AEDs will experience an ADR [4,5]. These studies have shown that the risk of an ADR is significantly greater in patients receiving polytherapy than in those receiving monotherapy [4]. Behavioural problems and somnolence were the most common ADRs [4].

Parents of children with epilepsy and the children and young people who are due to receive AED treatment have the right to know the likelihood of them experiencing an ADR, the types of ADRs they are likely to experience alongside when they should contact a health professional if they suspect they are experiencing an ADR. For example, patients due to commence treatment with carbamazepine or lamotrigine (especially lamotrigine in combination with valproic acid) should be warned of the risk of developing an allergic reaction. Allergic reactions are not uncommon with either carbamazepine or lamotrigine, but can lead on to Stevens-Johnson syndrome, which can be fatal [6]. Parents and older children should be informed that if they develop a rash that they should contact a health professional that day. Treatment should then be stopped as the prognosis for the onset of Stevens-Johnson syndrome or toxic epidermal necrolysis is poor. An alternative AED should be commenced a few days after the withdrawal of the offending drug.

Clinical trials are the gold standard for evaluating the effectiveness of medication. They are, however, poor at detecting ADRs. RCTs involving children with epilepsy and treatment with AEDs are particularly poor in that data for children is rarely reported separately [7]. A similar problem occurs in safety studies where data for children are not usually reported separately [8,9]. It is important to report safety data for children separately as they are more prone to certain ADRs, e.g., valproate hepatotoxicity is most likely in children under the age of seven years [10]. Prospective cohort studies are the best way of evaluating AED toxicity. Unfortunately, the prospective cohort studies that have been performed have all involved relatively small numbers of children (102–392) [4,5,11,12,13]. A multicentre prospective AED study is planned in the UK with the aim of recruiting 3000 children [14]. Such a study would be useful in providing evidence regarding comparative toxicity of AEDs.

Systematic reviews of the toxicity of individual AEDs have been useful in determining the likelihood of specific toxicity. A systematic review of lamotrigine suggested that rash occurred in 7.3% of patients [15]. Discontinuation of therapy was required in 1.9% of children receiving lamotrigine [15]. Headache, somnolence, nausea, vomiting, dizziness, and abdominal pain were all significantly lower when children received lamotrigine as a monotherapy [15]. A systematic review of levetiracetam toxicity identified behavioural problems and somnolence as the most common ADRs [16]. Again, drug toxicity was significantly more likely with polytherapy than with monotherapy. Discontinuation of the drug was necessary in 4.5% of children on polytherapy, but only 0.9% children on monotherapy [16].

A single prospective cohort study suggested that the discontinuation rate following treatment with either carbamazepine, valproic acid, or lamotrigine was 10% [4]. Due to the small numbers of patients in the studies, larger prospective cohort studies are essential in order to clearly establish the risk of drug toxicity. AEDs are essential for the management of epilepsy, but there remains little information regarding the comparative toxicity of individual AEDs. A comprehensive review of adverse cognitive and behavioural ADRs in children by the ILAE Task Force suggested that behavioural and cognitive ADRs were reported with many AEDs [17]. They were concerned, however, at the lack of good data for even the older AEDs, such as valproate. With epilepsy being the commonest neurological disorder in children, researchers have a responsibility to improve our understanding of AED toxicity in children. Parents of young children, and children themselves, have the right to know the risk-benefit of individual AEDs to ensure that they are actively involved in the discussions regarding treatment.
